# Dragon (repulsive guidance molecule b, RGMb) is a novel gene that promotes colorectal cancer growth

**DOI:** 10.18632/oncotarget.4110

**Published:** 2015-05-12

**Authors:** Ying Shi, Guo-Bin Chen, Xiao-Xiao Huang, Chuan-Xing Xiao, Huan-Huan Wang, Ye-Sen Li, Jin-Fang Zhang, Shao Li, Yin Xia, Jian-Lin Ren, Bayasi Guleng

**Affiliations:** ^1^ Department of Gastroenterology, Zhongshan Hospital, Xiamen University, Xiamen, Fujian Province, China; ^2^ Faculty of Clinical Medicine, Medical College, Xiamen University, Xiamen, Fujian Province, China; ^3^ State Key Laboratory of Cellular Stress Biology, Xiamen University, Xiamen, Fujian Province, China; ^4^ School of Biomedical Sciences, Faculty of Medicine, The Chinese University of Hong Kong, Shatin, N.T., Hong Kong, China; ^5^ School of Biomedical Sciences Core Laboratory, The Chinese University of Hong Kong Shenzhen Research Institute, Shenzhen, China; ^6^ Department of Nuclear Medicine, The First Affiliated Hospital of Xiamen University, Xiamen, Fujian Province, China; ^7^ Center for Molecular Imaging and Translational Medicine, School of Public Health, Xiamen University, Xiamen, Fujian Province, China; ^8^ MOE Key Laboratory of Bioinformatics, Tsinghua University, Beijing, China

**Keywords:** dragon, colorectal cancer, BMP, Smad1/5/8, Erk1/2, proliferation

## Abstract

Colorectal cancer (CRC) is one of the most commonly diagnosed cancers and a major cause of cancer death. However, the molecular mechanisms underlying CRC initiation, growth and metastasis are poorly understood. Dragon (RGMb), a member of the repulsive guidance molecule (RGM) family, has been recently identified as a co-receptor for bone morphogenetic protein (BMP) signaling, but the role of Dragon in CRC development is undefined. Here, we show that Dragon expression was increased in colon cancer tissues compared to control tissues in CAC mouse model and in human patients. Dragon promoted proliferation of CT26.WT and CMT93 colon cancer cells and accelerated tumor growth in the xenograft mouse model. Dragon's action on colon cancer development was mediated via the BMP4-Smad1/5/8 and Erk1/2 pathways. Therefore, our results have revealed that Dragon is a novel gene that promotes CRC growth through the BMP pathway. Dragon may be exploited as a potential therapeutic target for CRC treatment.

## INTRODUCTION

The colon/rectum (colorectum) is one of the most common cancer sites, and colorectal cancer (CRC) is a major cause of cancer-associated morbidity and mortality [1]. CRC develops from the normal colonic mucosa, which progressively undergoes hyperplasia, adenoma, carcinoma, invasion and metastasis [2]. Although the morbidity and mortality of CRC patients have been declining during the last decade according to the *Annual Report to the Nation on the Status of Cancer* [1], CRC is still the second leading cause of cancer deaths worldwide [3]. Molecular mechanisms underlying CRC formation and development are not fully understood. Therefore, novel biomarkers and targeting genes for CRC prediction and treatment remain to be identified. Previous studies have suggested that bone morphogenetic protein (BMP) signaling may play a role in CRC development [4]. However, the precise role of BMP signaling and its regulation in CRC are largely unknown.

BMPs are members of the TGF-β superfamily, which also comprises TGF-βs, activins and growth and differentiation factors (GDF). Canonical BMP signaling starts with binding of BMP ligands to the types I (ALK) and II (BMP-RII) receptors on the cell surface to activate the downstream receptor-regulated Smad proteins, i.e., R-Smads 1, 5 and 8. Activated R- Smads associate with the Co-Smad (Smad4) for translocation into the nucleus to regulate the transcription of target genes [5].

A member of the repulsive guidance molecule (RGM) family, Dragon (RGMb) is a co-receptor for BMP signaling [6-8]. Dragon was first identified in the dorsal root ganglion (DRG) [9]. However, Dragon is expressed not only in the embryonic and developing nervous system, but also in the epithelial cells of kidney tubules, where it enhances BMP4 signaling [10]. Our recent study demonstrated that Dragon inhibits E-cadherin expression in renal tubular cells in injured kidneys [11]. Dragon has also been shown to regulate macrophage function via the p38 and Erk1/2 MAPK pathways but not the Smad1/5/8 pathway [12]. In addition, Dragon interacts with neogenin, a receptor for the RGM family members, thus controlling aggregation and migration of neogenin-positive cells *in vitro* and *in vivo* [13-15]. However, the role of Dragon in the development of malignant diseases, especially in gastrointestinal cancers, remains to be identified. In the present study, we found that Dragon is up-regulated in colon cancer tissues and Dragon expression increases with CRC progression. Dragon promotes colon cancer cell proliferation and tumor growth via the Smad1/5/8 and Erk1/2 signaling pathways. Dragon-mediated colon cancer cell proliferation is dependent on BMP4.

## RESULTS

### Dragon expression is increased in colon cancer tissues

**Figure 1:**
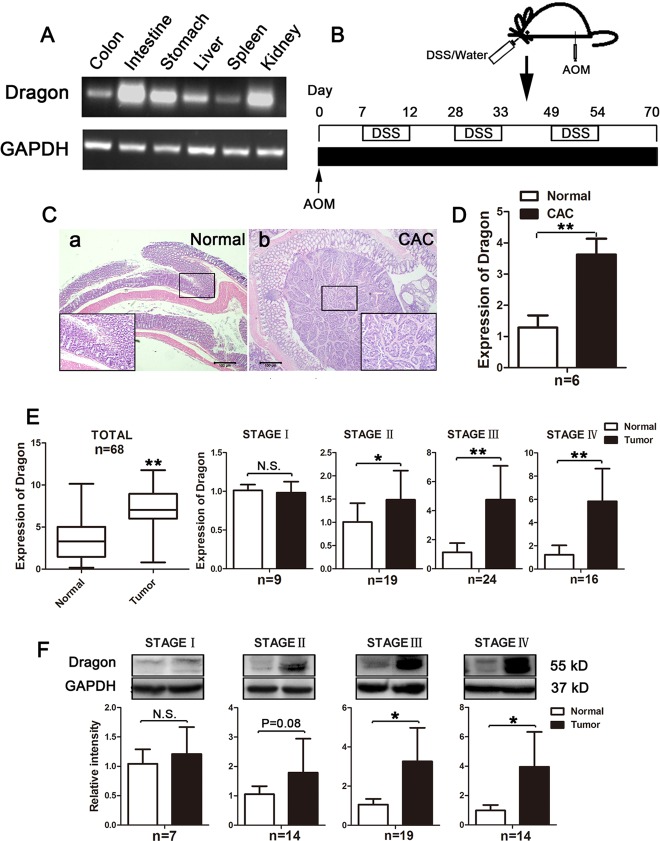
Expression of Dragon in normal colons and colorectal cancer tissues in mice and in human patients **A.** Dragon mRNA expression in different organs of normal mice. Total RNA was extracted from the colon, intestine, stomach, liver, spleen and kidney for RT-PCR to determine Dragon mRNA expression. GAPDH was used as a control. **B.** Experimental protocol for CAC induction. Mice were injected intraperitoneally with AOM at a dose of 12.5 mg/kg body weight. One week after the injection, 3% DSS was administered to mice via their drinking water for 5 days, and they were then switched to normal drinking water for 16 days. The treatments were repeated for three cycles. **C.** Hematoxylin-Eosin staining of the normal colon a. and CAC lesion b. **D.** Expression of Dragon mRNA in colon cancer tissues. Real-time PCR analysis was performed to determine Dragon expression in normal colons and colon cancer tissues. Tissues from 3 different sites of the normal colon or colon cancer were used for each mouse (*n* = 6, *P < 0.05). **E.** and **F.** Dragon mRNA and protein expression in tumor lesions collected from human CRC patients were analyzed. Normal tissues at the distance of 5 cm from the surgical margins were used as controls. (**E.**, left panel 1) Dragon mRNA expression in human colorectal cancer lesions and normal tissues. 68 patients were used. (**E.**, right panels) Dragon mRNA expression in human colorectal cancer lesions at different stages. Colorectal tumor tissues and normal tissues collected at stage I (*n* = 9), II (*n* = 19), III (*n* = 24) and IV (*n* = 16) were used to measure Dragon mRNA levels. **F.** Dragon protein expression in human colorectal cancer lesions at different stages. Colorectal tumor tissues and normal tissues collected at stage I (*n* = 7), II (*n* = 14), III (*n* = 19) and IV (*n* = 14) were used to measure Dragon protein by Western blotting. The upper panels are representatives of the Western blots at different stages, and the lower panels are densitometric analyses of the Western blots at corresponding stages. N.S., not significantly different; *P < 0.05; **P < 0.01.

We first analyzed Dragon expression in different organs in normal mice by RT-PCR. As shown in Figure 1A, Dragon mRNA was expressed in the colon although the expression levels were not as high as those in the stomach, intestine and kidney.

We then induced colitis-associated colorectal carcinoma (CAC) in mice using the protocol illustrated in Figure 1B. The CAC lesions were confirmed by Hematoxylin-Eosin staining (Figure 1C). Interestingly, Dragon mRNA was dramatically up-regulated in colon cancer tissues compared with normal colon tissues (Figure 1D).

We performed immunohistochemistry on human colorectal sections to determine the cell types that express Dragon. Dragon protein was localized to the glandular epithelium of the para-cancerous colon tissues (Supplemental Figures 1A), and Dragon expression was increased in cancer lesions compared to the para-cancerous colon tissues (Supplementary Figures 1B). These results suggest that Dragon expression is elevated in mouse and human colon cancer tissues.

We also examined expression of the two other RGM family members, i.e., RGMa and RGMc, and found that RGMa and RGMc (HJV) mRNAs were barely detectable in both normal colons and on CAC colons, and their expression levels were much lower than those of Dragon (Supplemental Figure 2).

### Dragon expression increases with CRC progression

To further analyze Dragon expression in human colon cancers, we collected tumor samples from 68 human CRC patients. Normal tissues at the distance of 5 cm from the surgical margins were used as controls. Dragon mRNA levels were significantly higher in human colorectal cancer lesions than in para-cancerous tissues (Figure 1E, panel 1). To correlate Dragon expression with CRC progression, we compared Dragon mRNA levels between colorectal tumors and control tissues at different stages. Dragon mRNA levels were similar between tumor tissues and control tissues at stage I, but they turned to be significantly higher in colorectal tumor tissues than in control tissues at stages II, III and IV (Figure 1E, panels 2-5).

Similar to the Dragon mRNA levels, Dragon protein levels were also significantly higher in colorectal cancer lesions than in control tissues at stages III and IV, whereas they were not different between cancer lesions and control tissues at stage I (Figure 1F). Dragon protein levels tended to be higher in colorectal cancer lesions than in control tissues at stage II (*P* = 0.08). Together, these results indicate that Dragon expression increases as CRC progresses.

### Dragon promotes colon cancer cell proliferation *in vitro*

**Figure 2:**
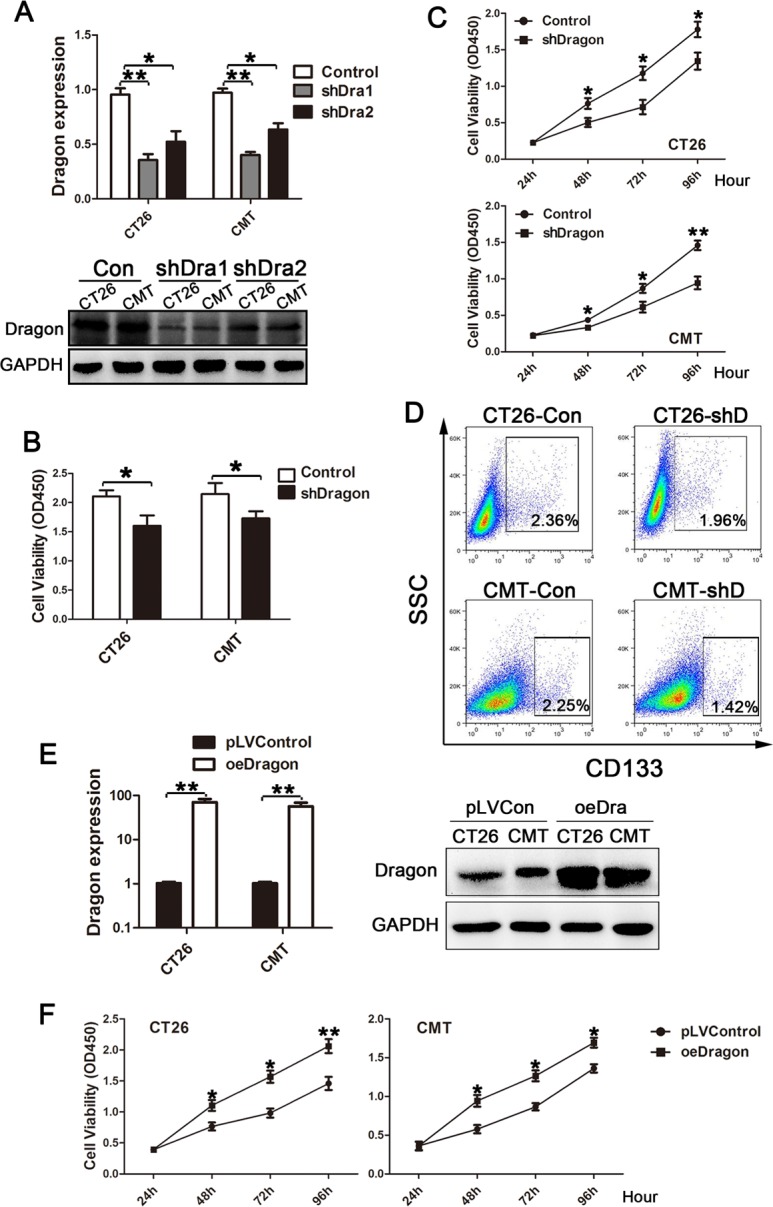
Effects of Dragon knockdown and overexpression on colon cancer cell proliferation **A.** Dragon expression in stable Dragon knockdown CT26.WT and CMT93 cells. Stable Dragon knockdown cells were generated from two Dragon shRNA sequences (shDra1 and shDra2). Whole lysates from Dragon knockdown and control CT26.WT and CMT93 cells were analyzed for Dragon mRNA levels by real-time PCR (upper panel) or Dragon protein by Western blotting (lower panel). **B.**, **C.** and **D.** Effects of Dragon knockdown on colon cancer cell proliferation. Dragon knockdown (shDragon or shD) and control CT26.WT and CMT93 cells were analyzed for cell viability using BrdU incorporation assay 24 h after cell seeding **B.** or CCK-8 cell proliferation assay from 24 to 96 h after cell seeding **C.**, or for CD133 expression using FCM analysis **D.**. Experiments were performed for 3 times independently. **E.** Dragon expression in stable Dragon overexpression (oeDra or oeDragon) CT26.WT and CMT93 cells. Stable Dragon overexpression CT26.WT and CMT93 cell lines were established, and cells transfected with empty pLV plasmid were used as controls. Whole lysates from Dragon overexpression and control CT26.WT and CMT93 cells were analyzed for Dragon mRNA levels by real-time PCR (left panel, *n* = 3) or for Dragon protein by Western blotting (right panel). **F.** Effects of Dragon overexpression on colon cancer cell proliferation. Dragon overexpression (oeDragon) and control CT26.WT and CMT93 cells were analyzed for cell proliferation using CCK-8 cell proliferation assays at 24, 48, 72 and 96 h after cell seeding. Experiments were performed for 3 times independently. *P < 0.05; **P < 0.01.

To determine the role of Dragon in the development of colon cancer, we established stable Dragon knockdown CT26.WT and CMT93 cell lines from two Dragon shRNA sequences (shDra1 and shDra2), as well as controls using the lentiviral system. Dragon expression was drastically inhibited in the Dragon knockdown CT26.WT and CMT93 cell lines at both mRNA (Figure 2A, upper panel) and protein (Figure 2A, lower panel) levels. Since shDra1 showed higher efficacy than shDra2 in inhibiting Dragon expression, we chose shDra1 for the subsequent experiments. We performed BrdU (Figure 2B) and CCK-8 (Figure 2C) proliferation assays and FCM analysis of CD133 expression (Figure 2D) on these cell lines to determine the effects of Dragon on cancer cell proliferation. All the three assays demonstrated that cell proliferation was slower in stable Dragon knockdown CT26.WT and CMT93 cells than in their respective controls. In addition, we performed Transwell migration and invasion assays, and found that cell migration and invasion were significantly inhibited in stable Dragon knockdown CT26.WT and CMT93 cells compared to the controls (Supplementary Figures 3A and 3B).

To corroborate the results from shRNA-mediated Dragon knockdown, we established stable Dragon over-expressing CT26.WT and CMT93 cell lines as well as pLV-controls. Dragon expression was drastically increased in the Dragon over-expressing CT26.WT and CMT93 cell lines at both the mRNA (Figure 2E, left panel) and protein (Figure 2E, right panel) levels. As shown by CCK-8 proliferation assays, stable Dragon-overexpressing CT26.WT and CMT93 cells exhibited an increased cell proliferation than their respective controls (Figure 2F). We also generated Dragon-overexpressed HCT116 human colon cancer cells (Supplementary Figure 4A). CCK-8 assay (Supplementary Figure 4B) and colony formation assay (Supplementary Figure 4C) showed that Dragon overexpression increased cell proliferation in HCT116 cells. These results collectively indicate that Dragon stimulates proliferation, migration and invasion of colon cancer cells.

### Dragon promotes tumorigenicity *in vivo*

**Figure 3:**
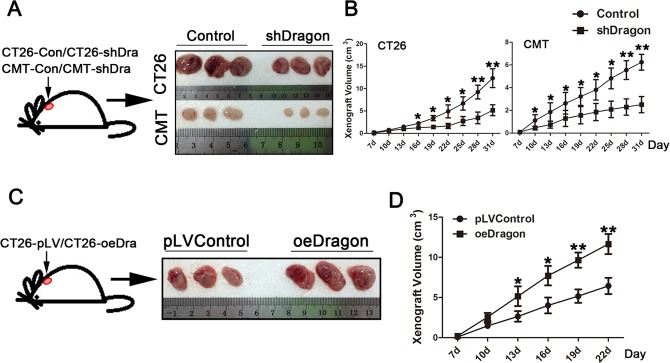
Effects of Dragon knockdown and overexpression on tumor growth in *xenograft* mouse model **A.** BALB/c mice were subcutaneously injected with control or stable Dragon knockdown (shDragon) CT26.WT cells. Mice were sacrificed at day 31 after cell injections. *Xenograft* tumors were isolated. **B.** BALB/c mice were subcutaneously injected with control or stable Dragon knockdown (shDragon) CT26.WT cells; and C57BL/6 mice were subcutaneously injected with control or stable Dragon knockdown (shDragon) CMT93 cells. Tumor sizes were measured at days 7, 10, 13, 16, 19, 22, 25, 28 and 31 after injections (*n* = 6). **C.** BALB/c mice were subcutaneously injected with Dragon overexpression (oeDragon) or control CT26.WT cells. Mice were sacrificed at day 22 after cell injections. *Xenograft* tumors were isolated. **D.** BALB/c mice were subcutaneously injected with Dragon overexpression (oeDragon) or control CT26.WT cells. Tumor sizes were measured at days 7, 10, 13, 16, 19 and 22 after injections (*n* = 6). *P < 0.05; * *P* < 0.01.

To examine whether Dragon plays a role in colon cancer development *in vivo*, we subcutaneously injected CT26.WT and CMT93 cells with or without stable Dragon knockdown into BALB/c and C57BL/6 mice. As shown in Figures 3A and 3B, xenografted tumor growth from stable Dragon knockdown cells was significantly slower than that from the control cells. To confirm the maintenance of Dragon knockdown efficiency, we sacrificed the mice at day 31 after cell injections and collected the tumor tissues for analysis of Dragon protein. Dragon protein levels were still significantly lower in the *xenografts* from stable Dragon knockdown cells than in those from control cells at day 31 after the injection (Supplementary Figures 5A and 5B).

We also subcutaneously injected CT26.WT cells with or without stable Dragon over-expression into BALB/c mice. As shown in Figures 3C and 3D, Dragon-overexpressing cells exhibited an increased tumor growth than the control cells. As expected, Dragon protein expression in the cancer cells was still dramatically up-regulated at day 22 after the injection (Supplementary Figure 5C). Together, these results suggest that Dragon promotes tumor growth *in vivo*.

### Dragon activates Smad1/5/8 and Erk1/2 in colon cancer cells

**Figure 4:**
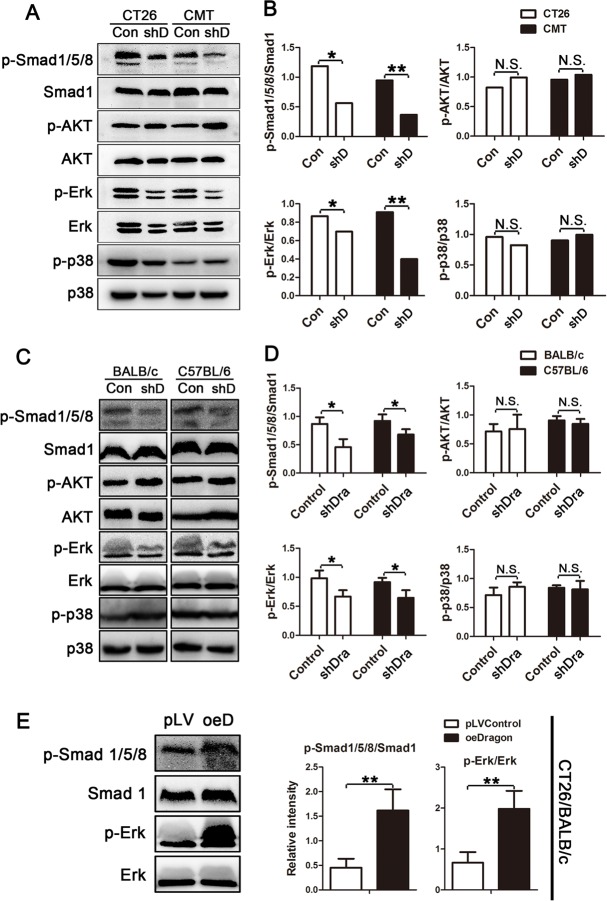
Effects of Dragon inhibition and overexpression on Smad1/5/8, AKT, Erk1/2 and p38 phosphorylation in colon cancer cells **A.** and **B.** Whole cell lysates from Dragon knockdown (shD) and control CT26.WT and CMT93 cells were used for Western blotting analysis **A.** for phospho-Smad1/5/8 (p-Smad1/5/8) and total Smad1 (Smad1); phospho-AKT (p-AKT) and total (AKT); phospho-Erk (p-Erk) and total Erk (Erk); and phospho-p38 (p-p38) and total p38 (p38). The Western blots were subjected to densitometric analysis **B.**. Experiments were performed for 3 times independently. **C.** and **D.** Lysates from xenografted tumors from Dragon knockdown (shD) and control CT26.WT and CMT93 cells collected from BALB/c and C57BL/6 mice respectively at day 31 after injections were used for Western blotting analysis for p-Smad1/5/8 and Smad1; p-AKT and AKT; p-Erk and Erk; and p-p38 and p38 **C.**. The Western blots were subjected to densitometric analysis **D.**; *n* = 6). **E.** Lysates from xenografted tumors from Dragon overexpression (oeDragon) and control CT26.WT cells collected at day 22 after injections in BALB/c mice were used for Western blotting analysis for p-Smad1/5/8 and Smad1; and p-Erk and Erk (right panel). Relative levels of p-Smad1/5/8 to Smad1, and p-Erk to Erk were obtained by densitometric analysis (left panel; *n* = 6). GAPDH is the loading control. N.S., not significantly different; *P < 0.05; ** *P* < 0.01.

To define the signaling pathways that mediate Dragon action on colorectal cancer cell proliferation, we analyzed Smad1/5/8, AKT, Erk1/2 and p38 phosphorylation levels in the stable Dragon knockdown CT26.WT and CMT93 mouse cells. Smad1/5/8 and Erk1/2 phosphorylation was down-regulated in the Dragon knockdown CT26.WT and CMT93 cells, while no significant differences in AKT and p38 phosphorylation were observed (Figures 4A and 4B).

We also examined Smad1/5/8, AKT, Erk1/2 and p38 phosphorylation levels in the tumors developed from Dragon knockdown and control CT26.WT and CMT93 cells in the *xenograft* model. Consistent with the results from cultured cells, Smad1/5/8 and Erk1/2 phosphorylation was down-regulated in the Dragon knockdown tumor tissues collected at day 31 after the injection, while AKT and p38 phosphorylation was not altered (Figures 4C and 4D). In addition, we observed increased Smad1/5/8 and Erk1/2 phosphorylation levels in xenografts from Dragon-overexpressing CT26.WT cells compared to control cells 22 days after the cell injection (Figure 4E). All these results suggest that Dragon activates Smad1/5/8 and Erk1/2 in colon cancer cells both *in vitro* and *in vivo*.

### Dragon promotes colon cancer cell proliferation via Smad1/5/8 and Erk1/2

**Figure 5:**
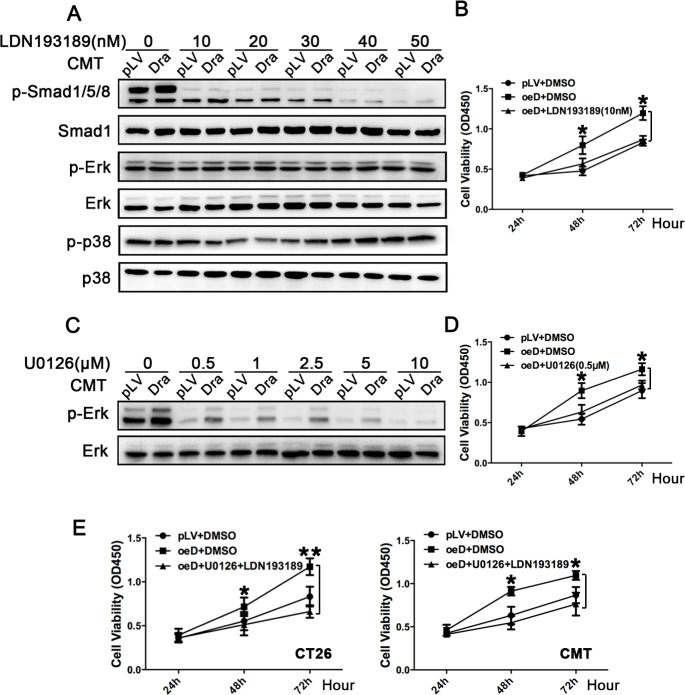
Effects of inhibition of the BMP-Smad1/5/8 and Erk1/2 pathways on colon cancer cell proliferation induced by Dragon **A.** and **B.** Effects of inhibition of the BMP-Smad1/5/8 pathway on Dragon-induced cell proliferation. Stable Dragon over-expression (Dra) and control (pLV) CMT93 cells were incubated for 1 h with and without increasing amounts of the BMP inhibitor LDN193189 (0-50 nM) before the cells were harvested for Western blotting analysis for phospho-Smad1/5/8, Smad1 phospho-Erk1/2, Erk1/2, phospho-p38 and p38 levels, **A.**. Control (pLV+DMSO), and Dragon overexpressing CMT93 cells in the absence (oeD+DMSO) or presence (oeD+LDN1923189) of LDN193189 at a dose of 10 nM were subjected to CCK-8 proliferation assay at 24, 48 and 72 h after cell seeding **B.**. Experiments were performed for 3 times independently. **C.** and **D.** Effects of inhibition of Erk activity on Dragon-induced cell proliferation. Stable Dragon over-expression (Dra) and control (pLV) CMT93 cells were incubated for 1 h with and without increasing amounts of the Erk inhibitor U0126 (0-10 μM) before the cells were harvested for Western blotting analysis for phospho-Erk and Erk levels **C.**. Control (pLV+DMSO), and Dragon overexpressing CMT93 cells in the absence (oeD+DMSO) or presence (oeD+U0126) of U0126 at a dose of 0.5 μM were subjected to CCK-8 proliferation assay at 24, 48 and 72 h after cell seeding **D.**. Experiments were performed for 3 times independently. **E.** Effects of inhibition of both the Erk1/2 and BMP-Smad1/5/8 pathways on Dragon-induced cell proliferation. Control (pLV+DMSO), and Dragon overexpression CT26.WT or CMT93 cells in the absence (oeD+DMSO) or presence (oeD+U0126+LDN1923189) of U0126 (0.5 μM) and LDN193189 (10 nM) were subjected to CCK-8 proliferation assay at 24, 48 and 72 h after cell seeding. Experiments were performed for 3 times independently. *P < 0.05, oeD+DMSO vs pLV+DMSO.

To determine whether Smad1/5/8 and Erk1/2 mediate Dragon's action on colon cancer cell proliferation, we used the BMP pathway inhibitor LDN193189 and the Erk1/2 inhibitor U0126. LDN193189 dose-dependently inhibited Smad1/5/8, but had no effect on Erk1/2 and p38 phosphorylation (Figure 5A), confirming the specificity of LDN193189 for Smad1/5/8. As expected, U0126 dose-dependently inhibited Erk1/2 phosphorylation (Figure 5C) phosphorylation. We incubated Dragon over-expressing CMT93 cells with 10 nM LDN193189, 0.5 μM U0126, or a combination of 0.5 μM U0126 and 10 nM LDN193189 before CCK-8 proliferation assays were performed (Figure 5B, 5D and 5E). The stimulation of cell proliferation by Dragon was inhibited by LDN193189 (Figure 5B), U0126 (Figure 5D), or U0126 plus LDN193189 (Figure 5E) in comparison with their respective controls. These results suggest that Dragon regulates colon cancer cell proliferation via the Smad1/5/8 and Erk1/2 pathways.

### Dragon-mediated colon cancer cell proliferation is dependent on BMP4

**Figure 6:**
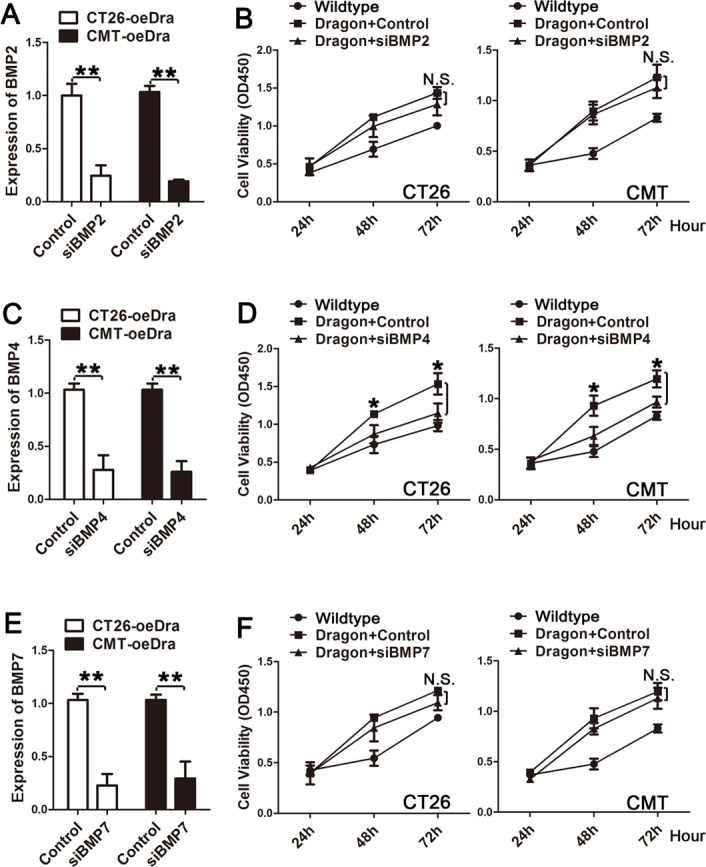
Effects of inhibition of BMP2, BMP4 or BMP7 expression on colon cancer cell proliferation induced by Dragon **A.** and **B.** Effects of inhibition of BMP2 on Dragon-mediated cell proliferation: **A.** Dragon overexpression (oeDra) CT26.WT or CMT93 cells were transfected with control siRNA or BMP2 siRNA (siBMP2). 48 h after transfection, cells were collected for real-time PCR analysis for BMP2 mRNA expression; **B.** Control or Dragon overexpressing CT26.WT (left panel) or CMT93 (right panels) cells transfected with or without BMP2 siRNA were subjected to CCK-8 proliferation assay at 24, 48 and 72 h after transfection. Experiments were performed for 3 times independently. **C.** and **D.** Effects of inhibition of BMP4 on Dragon-mediated cell proliferation: **C.** Dragon overexpression (oeDra) CT26.WT or CMT93 cells were transfected with control siRNA or BMP4 siRNA (siBMP4). 48 h after transfection, cells were collected for real-time PCR analysis for BMP4 mRNA expression; **D.** Control or Dragon overexpression CT26.WT (left panel) or CMT93 (right panels) cells transfected with or without BMP4 siRNA were subjected to CCK-8 proliferation assay at 24, 48 and 72 h after transfection. Experiments were performed for 3 times independently. **E.** and **F.** Effects of inhibition of BMP7 on Dragon-mediated cell proliferation: **E.** Dragon overexpression (oeDra) CT26.WT or CMT93 cells were transfected with control siRNA or BMP7 siRNA (siBMP2). 48 h after transfection, cells were collected for real-time PCR analysis for BMP7 mRNA expression; **F.** Control or Dragon overexpression CT26.WT (left panel) or CMT93 (right panels) cells transfected with or without BMP7 siRNA were subjected to CCK-8 proliferation assay at 24, 48 and 72 h after transfection. Experiments were performed for 3 times independently. N.S., not significantly different (Dragon vs Dragon+siBMP2 or siBMP7); *P < 0.05 (Dragon vs Dragon+siBMP4); **P < 0.01.

Previous studies have shown that Dragon is a co-receptor that enhances BMP2 and BMP4 signaling [16-19]. To determine whether Dragon's action on colon cancer requires BMP2 and BMP4, we first examined BMP2 and BMP4 expression in CT26.WT and CMT93 cells. We also included BMP7 as a control. As shown in Supplementary Figure 6, BMP4 and BMP7 were highly expressed in the two cell lines, while BMP2 was barely detectable. We then knocked down BMP2, BMP4 or BMP7 in the stable Dragon over-expressing CT26.WT and CMT93 cells. Transfection of BMP2 (Figure 6A), BMP4 (Figure 6C) or BMP7 (Figure 6E) siRNA reduced the respective mRNA expression by more than 75%. In the presence of control siRNA, Dragon over-expression promoted proliferation of CT26.WT and CMT93 cells (Figures 6B, 6D and 6F). Inhibition of BMP4 expression diminished the stimulation of proliferation of CT26.WT and CMT93 cells by Dragon (Figure 6D), while inhibition of BMP2 or BMP7 expression had no effect (Figures 6B and 6F). Previous studies have shown that BMP2 is a ligand for the Dragon co-receptor [16, 18]. The failure of inhibition of BMP2 expression to affect Dragon-mediated cell proliferation is unexpected, but it is most likely due to the low levels of endogenous BMP2 expression. Nevertheless, the results indicate that Dragon action on colon cancer cell proliferation is BMP ligand dependent.

**Figure 7:**
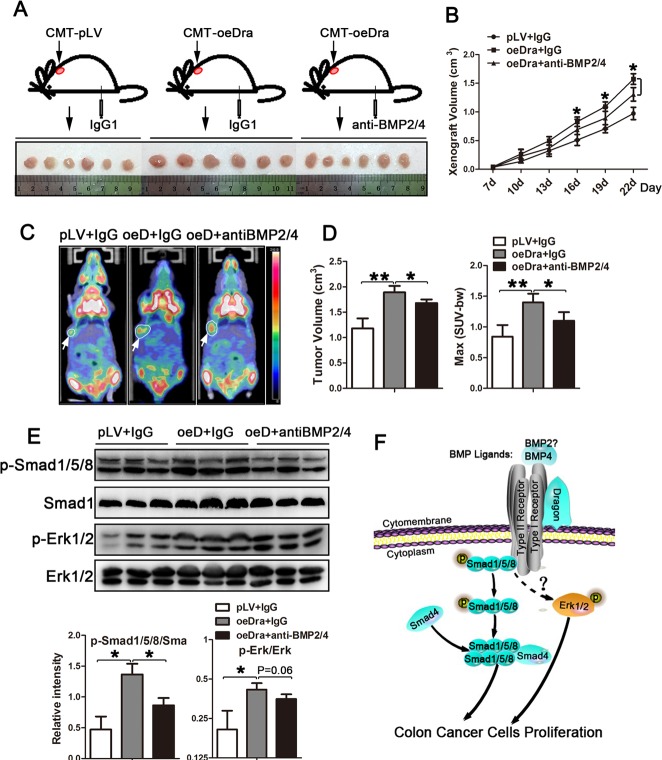
Effects of neutralization of BMP2 and BMP4 on xenografted tumor growth induced by Dragon CMT93 cells with or without Dragon over-expression were injected into C57BL/6 mice, followed by intraperitoneal injection of 2 μg/body weight of BMP2/4 antibody or IgG1 once every 2 days from the day of cell injection to 22 days after cell injection. **A.** *Xenografted* tumors were isolated from mice at 22 days after cell injection. **B.** The sizes of *xenografted* tumors were measured at days 7, 10, 13, 16 19 and 22 after cell injection (*n* = 6). *P* < 0.05, oeDra+anti-BMP2/4 vs oeDra+IgG. **C.** Representative images from Micro-PET/CT performed on mice at day 20 after cell injection: white circular outlines indicate *xenografted* tumors; arrows indicate SUV-MAX absorbance. **D.** Tumor volumes and SUV-MAX absorbance are quantified by Micro-PET/CT at day 20 after cell injection (*n* = 6). **E.** Phosphorylation levels of Erk1/2 and Smad1/5/8 in tumors collected at day 22 after cell injections (upper panel, Western blots; lower panel, densitometric analysis). **F.** Schematic diagram depicting Dragon action in regulating proliferation in colon cancer cells. Dragon/BMPs activate the Smad1/5/8 and Erk1/2 pathways via a mechanism yet to be determined and then promote colon cancer cell proliferation and tumor growth. *P < 0.05, **P < 0.01.

To examine whether BMP ligands are required by Dragon to induce tumor growth *in vivo*, we subcutaneously injected CMT93 cells with or without stable Dragon over-expression into C57BL/6 mice, followed by intraperitoneal injection of anti-BMP2/4 antibody or IgG at a dose of 2 μg/g body weight once every 2 days (Figure 7A). Anti-BMP2/4 antibody significantly suppressed xenografted tumor growth induced by Dragon overexpression (Figures 7A and 7B). Consistently, micro-PET/CT analysis showed that the sizes and Max SUV of xenografted tumors 20 days after cell injections were stimulated by Dragon, and this stimulation was inhibited by anti-BMP2/4 antibody (Figure 7C and 7D). Coupled with the changes in tumor sizes, Smad1/5/8 phosphorylation induced by Dragon in tumor tissues at day 22 was inhibited by anti-BMP2/4 antibody (Figure 7E). Erk1/2 phosphorylation induced by Dragon tended to be inhibited by anti-BMP2/4 antibody although the differences did not reach statistical significance (*P* = 0.06, Figure 7E). These results suggest that tumor growth induced by Dragon requires BMP ligands *in vivo*.

## DISCUSSION

RGM family contains four members, i.e., RGMa, RGMb, RGMc and RGMd. RGMa (RGM) and RGMb (Dragon) are expressed in the developing and adult central nervous system in distinct patterns [20]. RGMc, also named hemojuvelin (HFE2/HJV), positively regulates hepcidin, a key regulator of systemic iron homeostasis [21]. RGMd is expressed in fish but not in mammals [22]. Our results showed that Dragon is highly expressed in the colon. Compared with Dragon, RGMa and RGMc were expressed at much lower levels in the normal colons and the CAC colons. Therefore, Dragon is a major BMP co-receptor in the colon.

Our recent study showed that Dragon is highly expressed in renal tubular epithelial cells where it inhibits E-cadherin expression and increases apoptosis induced by hypoxia [11]. In addition, Dragon inhibits IL-6 expression in macrophages [12]. However, it is unknown whether Dragon plays a role in the gastrointestinal tract. RGMb is found to be one of the Myc-synthetic lethal genes and contributes to the Myc-driven human tumorigenesis [23]. Moreover, MYC gene is ranked at 9 (0.062%) among 14433 human genes for colorectal cancer resulting from a state-of-the-art disease gene prediction tool, CIPHER [24]. These analyses imply that RGMb may have a role in promoting colorectal cancer growth. Consistent with our previous study showing epithelial expression of Dragon in the kidney [10, 25], our present study revealed that Dragon was localized to the epithelial layer of colon. Dragon expression was increased in colon cancer lesions compared with normal or para-cancerous tissue in both the AOM/DSS-induced CAC mouse model and the CRC human patients. More importantly, our results showed that Dragon promoted colon cancer cell proliferation and CRC progression. Therefore, we reveal for the first time that Dragon is an oncogene that promotes colon cancer growth.

There are three phases involved in CRC development: initiation, promotion and progression. Our results from human patients showed that Dragon expression did not change in colon cancers at stage I compared to control tissues, but increased in colon cancers at stages II, III and IV. These results suggest that Dragon may not be involved in colon cancer initiation, but instead Dragon only promotes colon cancer growth.

Epithelial layer acts as a barrier to prevent harmful factors from gaining access to essential organs. Disrupted polarity and integrity of the epithelial layer impair its protective function, facilitating luminal epithelial cells to undergo transformation and migration [26]. Our previous study found that Dragon inhibits E-cadherin expression in IMCD3 renal tubular epithelial cells and in PNAC1 human pancreatic epithelioid carcinoma cells [11]. Therefore, it would be interesting to examine whether increased Dragon expression in colon cancer tissues inhibits E-cadherin expression, thus disrupting the epithelial integrity and promoting colon cancer metastasis.

BMPs act in an autocrine and paracrine manner [27]. Dysregulation of BMP signaling may contribute to development and progression of carcinoma [28, 29]. It has been reported that BMP6 promotes tumor proliferation through IL-10-dependent M2 polarization of tumor-associated macrophages in renal carcinoma [30]. BMP2 and BMP6 regulate hepatocellular carcinoma progression and prognosis [31, 32]. BMP6 and BMP9 affect the bone metastasis of prostate cancer and breast cancer respectively [33, 34]. Genetic variations in BMPs have been found in CRC patients [28, 35]. Dragon directly interacts with BMP ligands and receptors, and acts as a co-receptor that potentiates BMP2 and BMP4 signaling by enhancing interaction of BMPs with receptors and activating intracellular signaling transduction by Smad proteins [8, 36]. In this study, inhibition of BMP4 expression abolished Dragon's ability to stimulate proliferation in CT26.WT or CMT93 cells. Neutralization of BMP2 and BMP4 in mice inhibited xenografted tumor growth induced by Dragon. Thus, our results indicate that regulation of CRC growth by Dragon is mediated by the BMP pathway, and is dependent on BMP4. In addition, since BMP2 expression is low in both CT26.WT and CMT93 cells, it is not surprising that inhibition of BMP2 expression did not alter Dragon-induced proliferation in the two cell lines. However, our previous studies have shown that BMP2 is also a ligand for Dragon [16, 18]. Therefore, it is possible that BMP2 is also required by Dragon to induce tumor growth *in vivo*.

Our results are consistent with a previous study showing that BMP signaling stimulated the growth of primary human colon carcinomas [37]. Interestingly, a recent study demonstrated that BMP2 inhibited the proliferation of human colorectal cancer cells using adenovirus infection and the piggyBac transposon-mediated stable BMP2 overexpression [38]. It is unknown what caused the discrepancy between these studies, but it is unclear from the study by Zhang and colleagues whether the exogenous BMP2 gene was expressed and whether Smad1/5/8 was activated in the infected cells [38].

BMP ligands signal through the type II and type I serine/threonine kinase receptors to activate both the canonical Smad pathway and non-canonical mitogen-activated protein kinase (MAPK) [27, 39]. MAPKs can induce Smad6/7 activation, and the cross-signaling between the Smad and MAPK pathways may affect the fate of carcinogenesis [5, 40]. In the present study, Smad1/5/8 and Erk1/2 phosphorylation was enhanced by Dragon expression both in cultured colon cancer cells and in *xenografts*. Inhibition of the Smad1/5/8 and Erk1/2 pathways attenuated Dragon-induced colon cancer cell proliferation. All these results demonstrate that Dragon acts through the Erk1/2 and Smad1/5/8 pathways to induce colon cancer proliferation.

In conclusion, our present study reveals that Dragon stimulates colon cancer growth via the BMP4 and the downstream Smad1/5/8 and Erk1/2 pathways both *in vitro* and *in vivo* (Figure 7F). Dragon may be used as a potential therapeutic target for treating colon cancer.

## MATERIALS AND METHODS

### Ethics statement

This study was approved by the Ethics Committee of Zhongshan Hospital, Xiamen University (Xiamen, Fujian Province, China) (No. 20081009). Written consent was obtained from all the participants. All procedures involving experimental animals were performed in accordance with protocols that were approved by the Committee for Animal Research of Xiamen University and complied with the Guide for the Care and Use of Laboratory Animals (NIH publication No. 86-23, revised in 1985).

### Cell culture

The CT26.WT and CMT93 cells (purchased from ATCC, Manassas, VA, USA) were cultured in RPMI 1640 and Dulbecco's modified Eagle's medium (DMEM) respectively, each supplemented with 10% fetal bovine serum (Life Technologies, Grand Island, NY, USA) and 1% penicillin G/streptomycin. Cells were cultured at 37°C in an atmosphere of 95% air and 5% CO_2_.

### Establishment of stable dragon knockdown and over-expression cells

shRNA sequence 1 (5′– GTACCAAGCTGTGACAGATGA–3′), and sequence 2 (5′–GCCCCAGCTGGTAACTCTATC–3′) for the *Rgmb* gene were selected using our own original algorithm. Supernatants containing lentiviral particles for shRNA-mediated knockdown of Dragon were purchased from GenePharma (Shanghai, China). The titers of lentiviral stocks were ≤1×10^8^ (TU)/ml. 100 μl of the lentiviral supernatant was added into CT26.WT and CMT93 cell cultures in the presence of Polybrene (GenePharma). CT26.WT and CMT93 cells that were stably infected with the supernatants containing lentiviral particles from the empty pLVvector were used negative controls (Control). CT26.WT, CMT93 and HCT116 cells that stably overexpress Dragon were established by selection with puromycin (2.5 μg/mL, InvivoGen, San Diego, CA, USA) for 4 weeks. The knockdown efficiency was determined by real-time PCR and Western blotting analysis.

### Measurement of mRNA expression

Total RNA was extracted from cells and tissues using TRIzol (Invitrogen, Carlsbad, CA, USA) according to the manufacturer's instructions. First-strand cDNA was synthesized using the RevertAid first strand cDNA synthesis kit (Thermo Scientific, Fermentas, Lithuania). Transcripts of mouse BMP2, BMP4, BMP7, and RGMb were amplified using the primers described previously [10, 41].

### Western blot analysis

Total proteins were extracted from cells and tissues using the Mammalian Cell Lysis Reagent (Thermo Scientific, Rockford, IL, USA). Equal amounts of total proteins were separated by 10% SDS-PAGE and then transferred to polyvinylidene ﬂuoride (PVDF) membranes. Primary antibodies against p44/42 MAPK (Erk1/2, #4695), phospho- p44/42 MAPK (Erk1/2, Thr202/Tyr204, #4370), AKT (#4691), phospho-AKT (Ser473, #4060), p38 MAPK (#9212), phospho-p38 MAPK (Thr180/Tyr182, #9211), Smad1 (D59D7, #6944) and phospho-smad1 (Ser463/465)/5 (Ser463/465)/8 (Ser426/428, #9511) were purchased from Cell Signaling Technology (Boston, MA, USA). Primary antibody against Dragon (AF3597) and anti-sheep secondary antibody (HAF016) was purchased from R&D Systems (Minneapolis, MN, USA).

### Immunohistochemical staining

Colorectal cancer tissue samples were formalin-fixed, paraffin-embedded and sectioned. Antigen was retrieved by Citrate Antigen Retrieval solution (Maxim, Fuzhou, China). Sections were treated with peroxidase and blocked with 10% donkey serum. The sections were incubated with the Dragon antibody (AF3630, R&D Systems, Minneapolis, MN, USA) overnight at 4°C. After washes, the sections were incubated with anti-goat secondary antibodies (HAF109, R&D Systems, Minneapolis, MN, USA) and DAB Detection Kit (Maxim, Fuzhou, China) before they were counterstained with hematoxylin.

### CCK-8 cell proliferation assay

Cells with stable Dragon overexpression or inhibition and the respective controls were seeded into 96-well plates at a density of 2×10^3^ cells/well, in the presence or absence of the Smad1/5/8 signaling inhibitor LDN193189 or the Erk1/2 signaling inhibitor U0126. After 24, 48, 72, 96 h or 5 d, 10 μl of Cell Counting Kit-8 solution (DoJinDo, Tokyo, Japan) was added into each well, and the cells were cultured for additional 4 h. The absorbance was measured using a microplate reader at a wavelength of 450 nm.

### BrdU cell proliferation assay

The BrdU assays were performed using a BrdU kit (Cell Signaling Technology, Boston, MA, USA) according to the manufacturer's instructions. Briefly, cells were seeded into 96-well plates at a density of 2×10^4^ cells/well and incubated for 48 h. BrdU was added, and the cells were cultured for additional 4 h before Fixing/Denaturing Solution was added. Detection antibody solution was added and incubated for 1 h. Cells were then incubated with HRP-conjugated secondary antibody solution, and followed by TMB Substrate. The absorbance was measured using a microplate reader at a wavelength of 450 nm.

### Flow cytometry (FCM) analysis

Cells were dissociated from the dish using TE buffer (Invitrogen, Carlsbad, CA, USA) and labeled with an anti-mouse CD133-PE antibody (eBioscience, San Diego, CA, USA) on ice for 30 min following the standard protocol. The cells were analyzed using a FACS Calibur ﬂow cytometer (San Jose, CA, USA). Summit software (FlowJo, Los Angeles, CA, USA) was used to determine the number of positive cells.

### Cell migration and invasion assays

Migration and invasion of the control and Dragon knockdown CT26.WT and CMT93 cells were tested using 24-well-sized polycarbonate filters with 8 μm porosity (Corning, NY, USA) and 24-well-sized fibronectin-coated polycarbonate filters with 8 μm porosity (BD Biosciences, Bedford, MA, USA) respectively. Cells were added into upper Transwell chambers, and incubated for 22 h and fixed with methyl alcohol. The cells on the upper surface of the filter were removed by wiping with a cotton swab. The cells on the lower surface of the filter were stained with basic violet and counted using a microscope.

### Colitis-associated colorectal cancer (CAC) model

BALB/c and C57BL/6 mice were purchased from the Laboratory Animal Center (Shanghai, China). The mice were maintained in a specific pathogen-free environment and were generally used between 6 and 8 weeks of age. Mice were intraperitoneally injected with 12.5 mg/kg body weight of AOM in 0.2 ml saline. One week after the AOM administration, 3% DSS was administered to the mice via their drinking water for 5 days, and they were then switched to normal drinking water for 16 days. This procedure was repeated for three cycles, and their colons were harvested for analysis at day 70.

### Animals and tumor xenograft implantation model

CT26.WT cells (5×10^6^; CT26.WT-shDragon, CT26.WT-Control) and CMT93 cells (5×10^6^; CMT93-shDragon, CMT93-Control) were subcutaneously injected into the BALB/c and C57BL/6 mice. 7 days later, solid tumors were observed in all of the mice that received cell injections. The size of each tumor and the body weight of each mouse were monitored and recorded once every 3 days using a Vernier caliper. The tumor volume was calculated as [length (mm) × width (mm)^2^]/2.

### BMP2/4 neutralization

C57BL/6 mice were subcutaneously injected with CMT93 cells with or without stable Dragon over-expression, followed by intraperitoneal injection of anti-BMP2/BMP4 antibody (MAB3552, R&D, Minneapolis, MN, USA) or IgG_1_ isotype control (MAB002, R&D, Minneapolis, MN, USA) at a dose of 2 μg/g body weight, once every 2 days, for up to 22 days.

### microPET/CT scans

Micro PET/CT scans were performed using an Inveon micro-PET/CT scanner (Siemens, USA). For static scans, the mice bearing CMT93 *xenografts* were injected with about 3.7-7.4 MBq (100-200 μCi) of [18F]FDG via the tail vein (*n* = 6 for each group). At 1h p.i., static micro-PET images (5 min) were obtained after whole body CT scan (6min). The micro-PET and CT images were generated separately and then fused using Inveon Research Workplace (Siemens, USA). The images were reconstructed using ordered subset expectation maximization with three-dimensional resolution recovery (OSEM 3D) with CT-based attenuation correction, and scatter correction. For data analysis, the region of interest (ROI) was manually drawn and covered the whole tumor on the CT images. This ROI was copied to the corresponding PET images. Similarly, a circular ROI was drawn on the muscle of the opposite leg of the mouse on the CT images and copied to the PET images. Maximum standardized uptake value (SUV (max)) of the tumor and muscle in the ROIs were recorded. The tumor-to-muscle (T/M) ratio was calculated by dividing the signal intensity of the tumor by that of the muscle.

### Statistical analysis

Statistical analysis was performed using SPSS 17.0 software (SPSS Inc., Chicago, IL, USA). Data between two groups were compared using the Student's *t*-test. All values are expressed as the means ± standard deviation (SD), and *P* < 0.05 was considered to be statistically significant. Graphs were generated using GraphPad Prism 5.0 (GraphPad Software Inc., La Jolla, CA, USA).

## SUPPLEMENTARY FIGURES


