# Supplementary material for: Dragon (repulsive guidance molecule b, RGMb) is a novel gene that promotes colorectal cancer growth
Source: Oncotarget. 2015 May 12;6(24):20540–54. doi: 10.18632/oncotarget.4110 (PMC4653024; doi:10.18632/oncotarget.4110)
Supplement: Supplementary file 1 [file oncotarget-06-20540-s001.pdf]

Dragon (repulsive guidance molecule b, RGMb) is a novel gene that promotes colorectal cancer growth

Supplementary Material

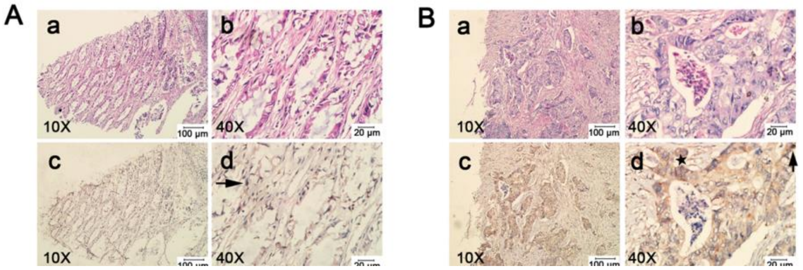

Supplementary Figure 1: Cellular localization of Dragon in the human colorectal cancer lesions and para-cancerous tissues by immunohistochemistry. All sections were stained with DAB (brown) and counterstained with hematoxylin (blue). Images are shown at both lower (×10) and higher (×40) magnifications. (**A**) Immunolocalization of Dragon in para-cancerous tissue (panels c and d). Adjacent sections were subjected to H.E. staining (panels a and b). (**B**) Immunolocalization of Dragon in colorectal cancer lesions (panels c and d). Adjacent sections were subjected to H.E. staining (panels a and b). Dragon is localized to the epithelial layer of colon. Dragon is more highly expressed in colorectal cancer lesions compared to para-cancerous tissues. Dragon is mainly found in the cytoplasm, and it is also expressed in cell membrane (pentagram) and nucleus (arrow).

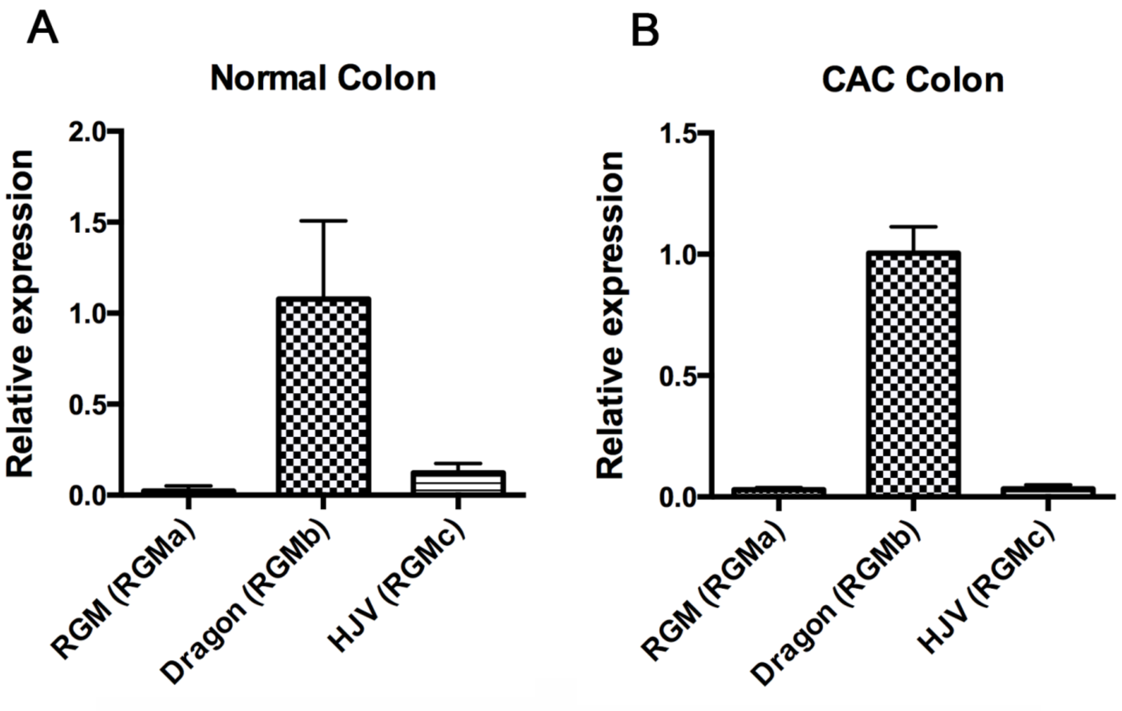

Supplementary Figure 2: Relative expression levels of RGMa, RGMb (Dragon) and RGMc (HJV) in normal colons (A) and the CAC colons (B) in mice. Real time PCR analysis was performed to measure RGMa, RGMb and RGMc mRNA levels.

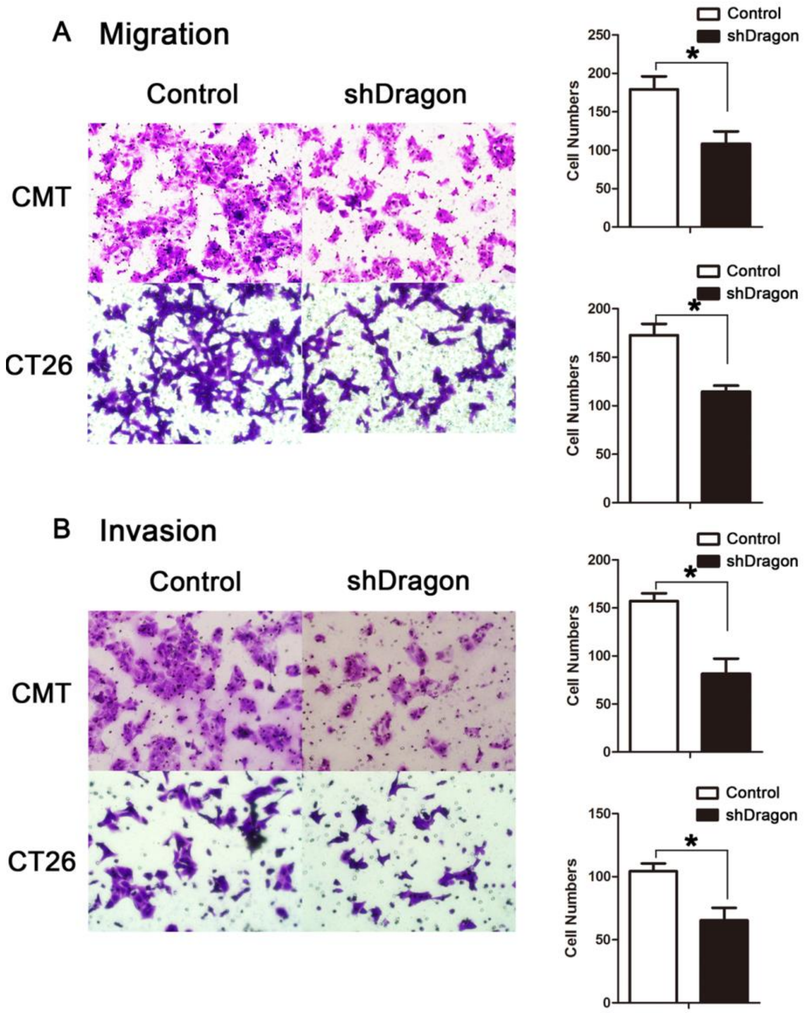

Supplementary Figure 3: Transwell migration and invasion assays. Inhibition of Dragon expression significantly decreased cell migration **(A and B)** and invasion **(C and D)** in both CMT93 and CT26.WT cells. The experiments were performed in triplicates. The results are quantitatively presented as histograms on the right, and representative images are shown on the left. Experiments were performed for 3 times independently. **P* < 0.05.

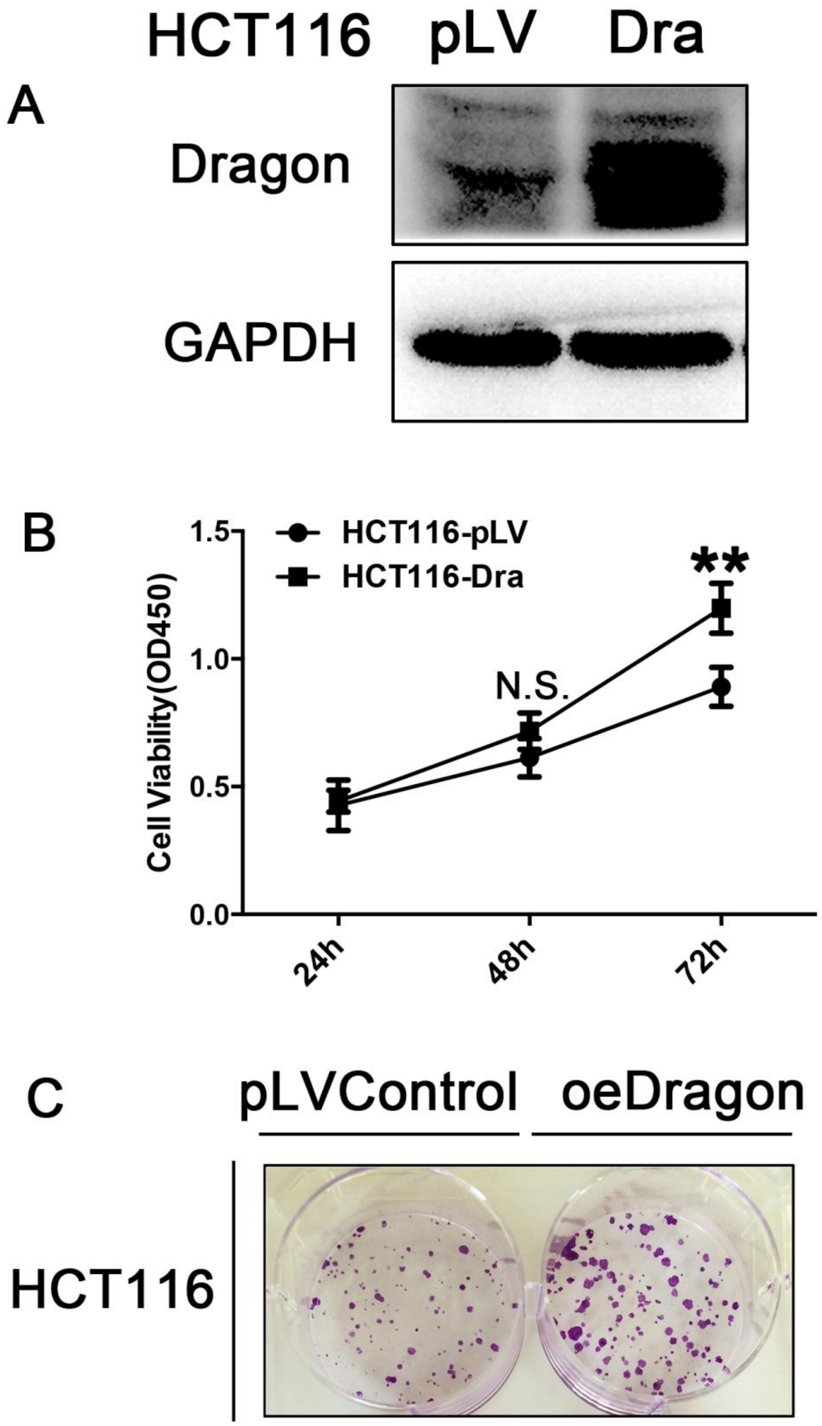

Supplementary Figure 4: Effects of Dragon overexpression on HCT116 proliferation. (**A**) Western blotting for Dragon in control (pLV) and Dragon over-expression (Dra) HCT116 human colon cancer cells. (**B**) Proliferation analysis. Dragon overexpression and control HCT116 cells were analyzed for cell proliferation using CCK-8 cell proliferation assays at 24, 48, 72 and 96 h after cell seeding. Experiments were performed for 3 times independently (***P* < 0.01). (**C**) Colony formation assays showed that Dragon overexpression increased the number of cell colonies (>50 cells) compared with pLV-control in HCT116 cells.

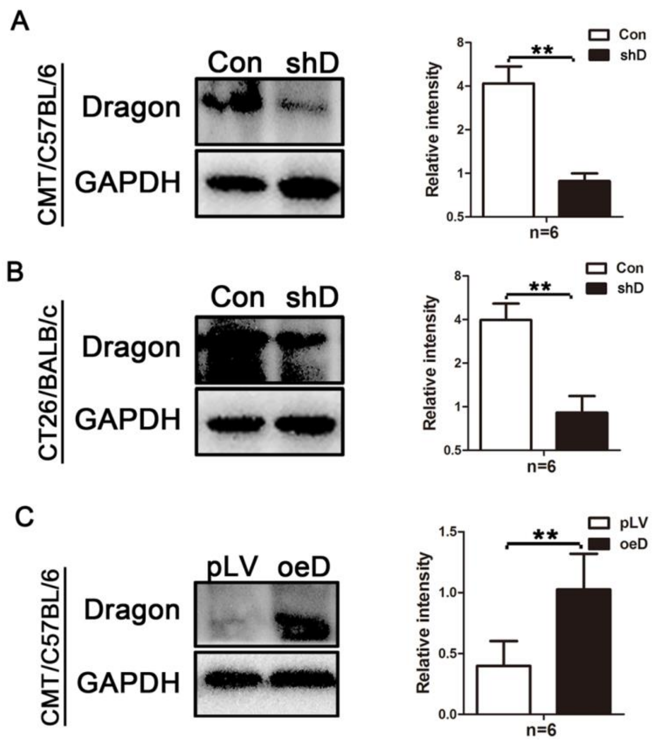

Supplementary Figure 5. (A and B) Dragon protein expression in tumors from Dragon knockdown (shD) and control CT26.WT (A) and CMT93 (B) cells collected at day 31 after cell injections (n=6). GAPDH is the loading control. ** *P* < 0.01. **(C)** Dragon protein expression in tumors from Dragon overexpressing (oeD) and pLV-control CMT93 cells collected at day 22 after injections (n=6). GAPDH is the loading control. ** *P* < 0.01.

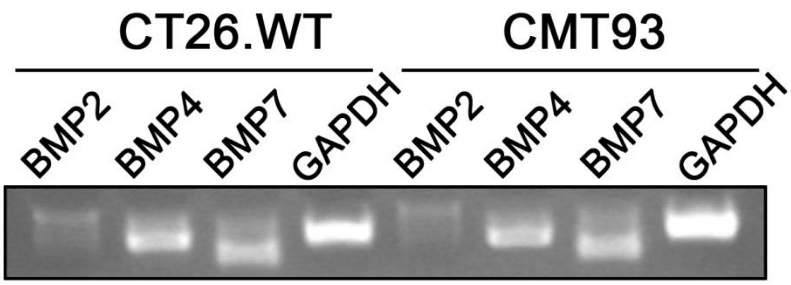

Supplementary Figure 6: BMP2, BMP4 and BMP7 mRNA expression in CT26.WT and CMT93 cells. Total RNA was extracted from CT26.WT and CMT93 cells to examine BMP2, BMP4 and BMP7 mRNA expression by RT-PCR. GAPDH was used as a control.
